# Oral Cyanocobalamin is Effective in the Treatment of Vitamin B12 Deficiency in Crohn’s Disease

**DOI:** 10.3390/nu9030308

**Published:** 2017-03-20

**Authors:** Fernando Gomollón, Carla J. Gargallo, Jose Fernando Muñoz, Raquel Vicente, Alberto Lue, Alberto Mir, Marta García-Alvarado, Marta Gracia, Santiago García-López

**Affiliations:** 1 Hospital Clínico Universitario “Lozano Blesa”, Avenue San Juan Bosco, 15, Zaragoza 50009, Aragón, Spain; carlajerusalen@hotmail.com (C.J.G.); alberto.lue@hotmail.com (A.L.); 2 Aragón Health Research Institute (IIS Aragón), Zaragoza 50009, Aragón, Spain; 3 University of Zaragoza, Zaragoza 50009, Aragón, Spain; 4 Centro de Investigación Biomédica en Red, Enfermedades Hepáticas y Digestivas (CIBEREHD), Madrid 28029, Spain; 5 Hospital Universitario de Salamanca, Salamanca 37007, Castilla y León, Spain; jmunozn@gmail.com; 6 Hospital Universitario Miguel Servet, Zaragoza 50009, Aragón, Spain; raquelvicentelidon@gmail.com (R.V.); tagaru@hotmail.com (M.G.); sgarcia.lopez@gmail.com (S.G.-L.); 7 Hospital Ernest Lluch Martin, Calatayud, Zaragoza 50299, Aragón, Spain; albertomirsubias@gmail.com; 8 Hospital Virgen de la Concha, Zamora 49022, Castilla y León, Spain; mery_271@hotmail.com

**Keywords:** vitamin B12 deficiency, cobalamin deficiency, Crohn’s disease, ileal resection, oral treatment, acute treatment, maintenance treatment

## Abstract

Cobalamin deficiency is common in patients with Crohn’s disease (CD). Intramuscular cobalamin continues to be the standard therapy for the deficiency and maintenance treatment in these patients, although oral route has been demonstrated to be effective in other pathologies with impaired absorption. Our aims were to evaluate the efficacy of oral therapy in the treatment of cobalamin deficiency and in long-term maintenance in patients with Crohn’s disease. We performed a multicenter retrospective cohort study that included 94 patients with Crohn’s disease and cobalamin deficiency. Seventy-six patients had B12 deficiency and 94.7% of them normalized their cobalamin levels with oral treatment. The most used dose was 1 mg/day, but there were no significant differences in treatment effectiveness depending on the dose used (≥1 mg/24 h vs. <1 mg/24 h). Eighty-two patients had previous documented B12 deficiency and were treated with oral B12 to maintain their correct cobalamin levels. After a mean follow-up of 3 years, the oral route was effective as maintenance treatment in 81.7% of patients. A lack of treatment adherence was admitted by 46.6% of patients in who the oral route failed. In conclusion, our study shows that oral cyanocobalamin provides effective acute and maintenance treatment for vitamin B12 deficiency caused by CD with or without ileum resection.

## 1. Introduction

Vitamin B12 (cobalamin) is a water-soluble vitamin that is essential for DNA synthesis, effective erythropoiesis, nervous system maintenance, and metabolism of protein, fat, and carbohydrate [1]. Humans cannot synthesize it, and hence need to obtain it through the diet, with it found almost exclusively in food of animal origin [2]. The manifestation of cobalamin deficiency ranges from subtle, non-specific clinical features to serious neurological and neuropsychiatric complications; because of that it is important to ensure an adequate intake and to provide a suitable screening for B12 deficiency in patients at risk [1]. A typical Western diet contributes 3–30 µg of B12 per day, of which 1–5 µg are absorbed, towards the recommended dietary allowance of 1 to 3 µg/day. Also, the body storage is relatively high (2000–5000 µg), which explains why clinical manifestations of B12 deficiency often appear late. Metabolism of this vitamin is complex and is made up of many processes, defects in any one of which can lead to deficiency [3].

Crohn’s disease (CD) is a chronic inflammatory gastrointestinal disorder that can affect any segment of the gastrointestinal tract, including the terminal ileum, where most of the absorption (98%) of B12 occurs. The reported prevalence of B12 deficiency in CD ranges from 5.6% to 38% and main causes of this deficiency are ileal resection and ileal disease (activity or fibrosis) [4,5,6,7,8,9,10,11].

The classical treatment for B12 deficiency is parenteral administration, in most countries intramuscular injection. However, a study performed in 1968 showed that 0.5% to 4% of radioactively labeled oral B12 can be absorbed along the entire intestine by passive diffusion in healthy people and in patients with pernicious anemia [12]. Although in normal conditions this may be a secondary mechanism, it may gain importance when the active mechanism mediated by intrinsic factor is affected. Several later randomized trials compared high oral doses (1000 to 2000 µg/day) with parenteral administration in patients with pernicious anemia, atrophic gastritis, or ileum or gastric surgery have shown that high daily doses of oral B12 have similar or better short-term improvements in B12 levels and symptoms resolution than parenteral administration [13,14,15]. In patients with CD, oral therapy may be as effective as parenteral, but is currently underexplored. Therefore, the aims of our study are: (1) to evaluate the efficacy of oral therapy in the treatment of vitamin B12 deficiency in patients with Crohn’s disease and (2) to analyze its efficacy in long-term maintenance treatment.

## 2. Materials and Methods

### 2.1. Definition of Terminology

The diagnosis of CD was performed in the patients included in our study according to the Lennard-Jones definition [16], which includes four groups of diagnosis criteria: clinical, radiological, endoscopic, and pathological criteria. The presence of at least two criteria is required and the pathology is the definitive one. Moreover, the sub-classification of Crohn’s disease by phenotype was performed according to Montreal classification [17] that considered age of onset (*A*; *A1*: *below 16 years old*, *A2*: *between 17–40 years old, and A3*: >*40 years old*), disease location (*L*; *L1*: *ileal*, *L2*: *colonic*, *L3*: *ileocolonic, and L4*: *isolated upper disease modifier*), and disease behavior (*B*; *B1*: *non-stricturing and non-penetrating*, *B2 stricturing*, *B3 penetrating, and p: perianal disease modifier*) as the predominant phenotypic elements.

The diagnosis of B12 deficiency was defined according to local laboratory criteria as serum cobalamin ≤200 pg/mL. Serum cobalamin concentrations were measured using chemiluminescence, with the assay from Beckman Coulter, which has a coefficient of variation <12% and a very good correlation with radioimmunoassay (r > 0.95).

Finally, the diagnosis of anemia and macrocytosis were performed according to international standards and local laboratory criteria. In adults, anemia is defined as a hemoglobin (Hb) levels below 12 gr/dL for non-pregnant women, 11 gr/dL for pregnant women, and 13 gr/dL for men [18] and macrocytosis is defined as the presence of macrocytes on a blood film, together with a raised mean corpuscular volume (MCV) (>98 fL) [18].

### 2.2. Study Population

This investigation is a cohort study with retrospective data collection conducted in four general hospitals integrated into the Spanish National Health System.

To address our aims, we distinguished two cohorts of patients: (1) patients with B12 deficiency and (2) patients with previous B12 deficiency who required maintenance treatment. Thus, to address our first aim, we included in our study patients attending our adult Inflammatory Bowel Disease Units diagnosed with CD and B12 deficiency who had been treated with B12 by oral route between January 2001 and November 2012. To address our second aim, we included patients attending our adult Inflammatory Bowel Disease Units diagnosed with CD and in who B12 deficiency was treated successfully with oral or intramuscular B12 between January 2001 and November 2012, but where maintenance treatment had then been conducted orally.

We included 94 patients with CD and B12 deficiency in our study, with a slight predominance of men. All patients were white and the average age of participants was 45 ± 13 years. Twenty-seven (28.7%) patients were active smokers. In relation to characteristics of CD at diagnosis of our patients, and according to Montreal Classification, more than 95% of them had disease in the terminal ileum (L1 or L3). Two (2/94) patients also had upper gastrointestinal disease and 11 (11/94) also had perineal disease. Finally, a surgical resection of the ileum had been performed in 24 patients (25.5%). Data about resection length were not available in all patients. Table 1 shows clinical and demographic characteristics of the study population.

Notably, none of the patients had neurological symptoms secondary to B12 deficiency. Also, none of the included patients had a previous diagnosis of pernicious anemia, and only one patient had prior history of gastric surgery.

### 2.3. Therapeutic Regimens

The different vitamin B12 oral treatment regimens used for treatment of B12 deficiency and for maintenance treatment are shown in Table 2 and Table 3*.*

### 2.4. Statistical Analysis

An initial exploratory analysis of all clinical variables was carried out. Continuous variables were expressed as the mean and standard deviation or median and interquartile range; whereas, qualitative variables were expressed as frequencies and percentages. The relationship between qualitative variables was evaluated with the chi-square test or the Fisher exact test if the expected effective cases were less than 5. The student *t*-test or Mann-Whitney U test was employed for comparing means of two independent groups. Normality was tested using the Kolmogorov-Smirnov test. For all tests, a two-sided *p*-value < 0.05 was considered statistically significant. The statistical analyses were performed using SPSS software v22.0 for Windows.

## 3. Results

### 3.1. Effectiveness of Oral Treatment in Vitamin B12 Deficiency

**Figure 1:**
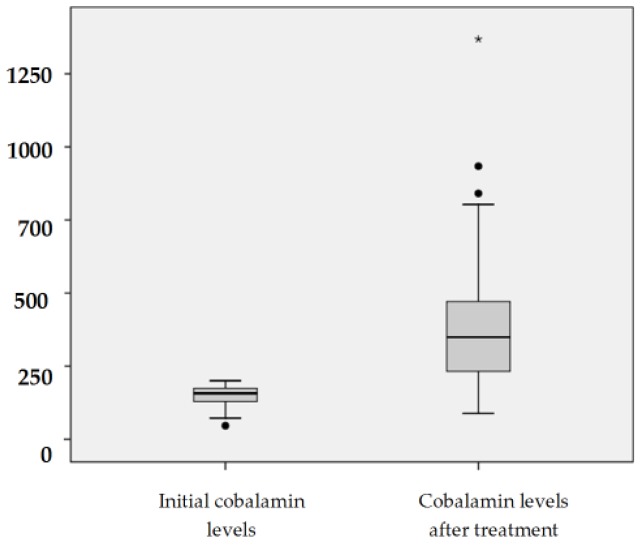
Serum cobalamin levels (pg/mL) before and after treatment with oral cyanocobalamin. * Extreme value (1369 pg/mL).

Seventy-six patients of the overall population included (94 patients) had vitamin B12 deficiency and were treated with oral B12 therapy to correct their deficiency. In these patients, the mean serum cobalamin concentration before oral treatment was 152.83 ± 34.09 pg/mL (median 157, interquartile range of 132–174.5 pg/mL). Seventy-two (94.7%) patients normalized their vitamin B12 levels (>200 pg/mL) after mean treatment duration of 193 ± 278 days (median 120, interquartile range 78–191). The mean serum cobalamin concentration after treatment increased to 398.14 ± 208.90 pg/mL (median 354.59, interquartile range: 242 to 474.50). Figure 1 shows the evident change of B12 levels and Table 4 cobalamin levels of each patient before and after treatment. In relation to hematologic manifestations of B12 deficiency, before oral B12 treatment, 19.7% (15) of patients had anemia (mean concentration of Hb: 13.61 ± 1.67), but no patient had macrocytic anemia. The erythrocyte MCV was elevated in 14 (18.4%) patients, although without anemia, with a mean of MCV of 91.54 ± 7.58. After treatment with oral vitamin B12, there were no significant changes in Hb concentration nor in MCV.

Folic acid levels (ng/mL), before and after treatment with oral cyanocobalamin, are shown in Supplementary Table 1 (Table S1).

In the subgroup of patients with ileal resection (21 patients), the mean serum cobalamin concentration before oral treatment was slightly lower than in patients without ileum surgery, but the differences were non-significant (151.43 ± 39.37 pg/mL vs. 153.36 ± 3.23 pg /mL, *p* > 0.05). Two of the four patients that did not normalize B12 levels after oral treatment had ileum resection. After oral B12 treatment, the mean serum cobalamin concentration increased significantly in patients with ileal resection and in patients without prior surgery (368.38 ± 189.45 pg/mL and 409.51 ± 216.42 pg/mL). There were no significant differences in the B12 levels reached between both groups.

Different oral treatment regimens were used; the daily dose of B12 used ranged from 0.004 to 3 mg, with 1 mg daily being the most used dose (in 53 patients, 69.7%). Fifty-nine patients (77.6%) were treated with doses of oral B12 ≥1 mg/day. There were no significant differences in oral treatment effectiveness in the normalization of B12 levels depending on the dose used (≥1 mg/24 h vs. <1 mg/24 h; 94.9% vs. 94.1%, *p* > 0.05). The different regimens used are shown in Table 2.

### 3.2. Effectiveness of Oral Route in Maintenance Treatment

**Figure 2:**
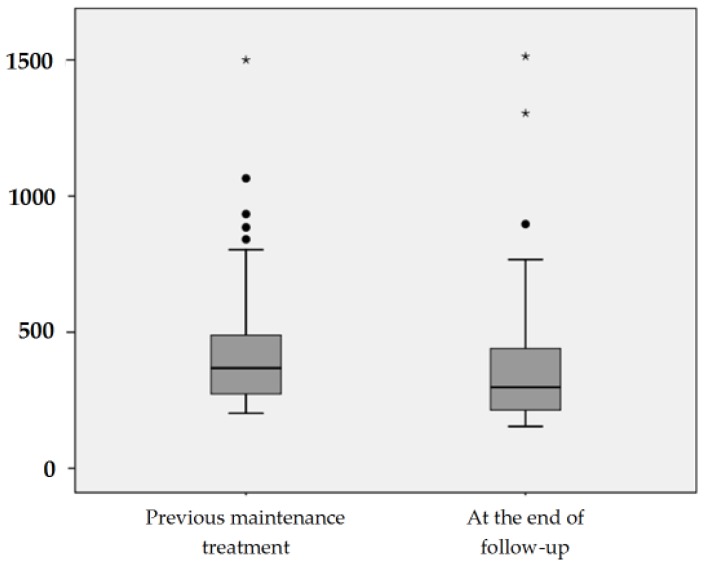
Serum cobalamin levels (pg/mL) during maintenance therapy; at the beginning of follow-up and at the end of follow-up. * Extreme values (1513 pg/mL, 1500 pg/mL and 1304 pg/mL, see Table 5).

Eighty-two patients with CD and previous documented B12 deficiency were treated with oral B12 to maintain their correct cobalamin levels. Seventy-eight percent of them (64/82) had been previously treated with oral B12 to correct the deficiency and 22% (18/82) with intramuscular B12 treatment. The mean serum cobalamin concentration before oral maintenance treatment was 429.39 ± 222.65 pg/mL (median 368, interquartile range of 273–489 pg/mL). After a mean follow-up of 1091 ± 841 days (median 892, interquartile range 375–1765), the oral B12 route was effective to keep serum cobalamin levels >200 pg/mL in 81.7% (67) of patients. The mean serum cobalamin concentration after follow-up was 364.45 ± 226.62 pg/mL (median 298.5, interquartile range of 214–440 pg/mL). See Figure 2 and Table 5. Interestingly, a lack of oral B12 treatment adherence was admitted by almost half (46.6%, 7/15) of the patients who failed to keep normal levels of cobalamin with the oral maintenance treatment.

The daily dose of B12 used as maintenance treatment ranged from 0.004 to 2.5 mg, with 2 mg weekly (0.28 mg/day) being the most used dose (in 40 patients, 48.7%). There were no significant differences in oral maintenance treatment effectiveness depending on the dose used (>0.28 mg/24 h vs. ≤0.28 mg/ 24 h; 83.4% vs. 80%, *p* > 0.05). The different oral treatment regimens used are shown in Table 5.

## 4. Discussion

The traditional treatment of vitamin B12 deficiency is intramuscular injection, however, this vitamin may be offered orally. The increasing evidence that supports the use of oral vitamin B12 as an effective alternative to parenteral administration prompted the spread of oral administration in some countries, such as Sweden or Canada. However, in the rest of the world, intramuscular route remains the most used. Indeed, controversy still surrounds the advantages and effectiveness of the oral therapy [19]. Three randomized controlled trials (RCTs) have compared oral and intramuscular vitamin B12 in patients with B12 deficiency from different causes and, although relatively short in duration (<4 months) and of small sample size (158 patients in total), showed that oral was as effective as intramuscular therapy in normalizing B12 levels and hematological alterations [13,14,15]. We want to emphasize that two of these RCTs excluded patients with inflammatory bowel disease [13,14] and the other included patients with CD, but the number was not shown in the publication [15]. Our large case series specifically evaluates oral administration route in CD and shows that oral cyanocobalamin provides effective acute and maintenance treatment for vitamin B12 deficiency caused by CD with or without ileum resection. Moreover, our follow-up periods are long, unlike previous studies. We found no significant changes in Hb concentration nor MCV after oral B12 therapy. It is important to note that, in our study, 20% of patients had anemia but none had macrocytic anemia, probably because most anemia was not attributable to B12 deficiency. In CD, cobalamin deficiency is rarely associated with anemia and most anemia is attributable to iron deficiency or chronic inflammation [5]. To our knowledge, there is a unique report that evaluated the efficacy of oral B12 replacement in CD. This study, which was published in 2014 in the “letters to the editor” section by Plener et al., concluded that 27 patients with low baseline values achieved normal serum B12 levels with oral B12 therapy and nine maintained them [20]. The data provided by authors are scarce. We want to note that, in 2013, we had presented the preliminary data for our study in the annual congress of Spanish Gastroenterology Association and also in Congress of European Crohn’s and Colitis Organization. Therefore, we believe that our study was the first in evaluating oral B12 therapy in Crohn’s disease patients [21,22].

In our opinion, it is necessary to know if oral treatment is effective in patients with CD. These patients may have significant portions of ileum affected by inflammation or resected and thus the active B12 absorption may have dramatically decreased and even passive absorption may be affected. Until now, most of the patients included in previous studies have had pernicious anemia, atrophic gastritis, gastric surgery, or low diet intake, ignoring patients with CD [13,14,15,23,24]. The possibility of taking an oral preparation would also allow patient preferences to be taken into account when deciding on what treatment to prescribe. Oral administration should provide patients with greater autonomy, improve treatment satisfaction, reduce treatment costs, and improve safety in anticoagulated patients [23,25,26,27]. Nyholm et al. showed that all patients included in their study found oral therapy to be acceptable or highly acceptable and that 87% preferred oral to intramuscular therapy [23]. A Canadian study of primary care found that 73% of patients taking intramuscular B12 were willing to do a trial switch to oral route and after 6 months, 71% wished to remain on oral treatment permanently [25]. In relation to cost, there is little difference in the cost of oral versus intramuscular therapy when the medication alone is considered. However, intramuscular administration often involves a special trip to a health facility or a home visit by a health professional to administer the injection. Oral treatment could therefore save considerable Health Service resources. For example, a Canadian study [27] estimated that converting patients aged over 65 years on B12 replacement from the intramuscular to oral form could save between $2.9 and $17.6 million (2.5 and 15.3 million Euro) over five years in Ontario alone. In our public National Health System, B12 ampules are covered under all provincial health drug plans, while B12 tablets are not covered. Thus, oral B12 tablets probably save public Health Service resources, but are more expensive for Spanish patients. Because of that, one of the most used formulations in our study for maintenance treatment is B12 ampules, but by oral route. In our opinion, all advantages and disadvantages of both administration routes must be evaluated and explained to the patient. Although oral administration seems to be easy, effective, and less costly than the intramuscular route, some patients may benefit from traditional administration. For example, intramuscular therapy may be a better option to ensure timely administration in patients with non-adherence to oral medication. In our study, we detected that 8.5% of our patients in maintenance treatment had low-adherence, which resulted in treatment failure. This low adherence could be explained by some characteristics of our population related to low treatment adherence in patients with CD: a population that includes many young adults, many that take multiples drugs and with asymptomatic B12 deficiency [28]. Briefly, evaluating risk factors for low adherence to the different routes of administration is important before choosing one of them.

The oral dosage to be used in patients with CD is not yet to established. In the three RCTs that evaluate the oral therapy [11,12,13] and in most case series [20,23,24], high oral doses (1000–2000 µg/day) were used, but in these studies very few patients with CD were included. Eussen et al. showed in their dose-finding trial that the lowest dose of oral vitamin B12 required to normalize mild deficiency in older people was more than 200 times greater than the recommended dietary intake (3 µg/day) [29]. In view of the estimated 1% of total absorption by passive diffusion, a 1000 µg daily dose is recommended in patients with pernicious anemia. However, patients with CD and/or short ileal resection probably maintain, although not intact, a percentage of their active B12 absorption, and thus lower doses may be sufficient, especially in maintenance treatment. In our study, 2 mg weekly (0.28 mg daily) for maintenance treatment was the most used dosage (48.7%) with high effectiveness. In fact, there were no significant differences between this dosage and other higher dosages. For treatment of the deficiency, we used high doses, with 1 mg being the most used dose (69.7%) but there were also no significant differences between this dosage and other lower dosages. Also, our patients had good tolerability without adverse events after high oral dose of vitamin B12.

Finally, the study’s strengths include its large sample size and long follow-up period that allow for the evaluation of long-term efficacy. We also acknowledge several limitations. It is a retrospective study that did not includeB12 deficient patients with neurological symptoms. Also, functional biomarkers of the deficiency (methylmalonic acid and homocysteine concentrations) were not taken into account as diagnostic markers and outcome variables because these are not normally determined in our country, either at diagnosis or during follow-up.

## 5. Conclusions

To our knowledge, the present study represents the first study that specifically evaluated the role of oral administration route in the treatment of B12 deficiency in patients with CD, and also is one of the largest cohorts of patients with B12 deficiency treated orally. According to our results, oral administration of cyanocobalamin must be strongly considered and may be even the regimen of choice to treat B12 deficiency in CD. It is effective as a means in which to treat deficiency and also as a maintenance treatment, being well tolerated in these patients. Other studies are still required to test if oral B12 is suitable in patients with neurological symptoms. The oral dosage to be used in these patients is not yet fully established, but probably moderately high doses (1 mg/day) are required for initial treatment and lower doses for maintenance treatment. A close monitoring with clinical review and repeat vitamin B12 levels may help to establish the most adequate maintenance doses. Patient preference is a factor of prime importance in the choice of administration route in order to avoid low treatment adherence. Finally, further research is needed to avoid perpetuating oral cyanocobalamin replacement as one of “medicine’s best kept secrets” [30].

## Figures and Tables

**Table 1:** Clinical and demographic characteristics of the study population.

Patients Demographics	Overall Study Population (94 Patients) *n* (%)	Cohort B12 Deficiency Treatment (76 Patients) *n* (%)	Cohort Maintenance Treatment (82 Patients) *n* (%)
**Sex (male)**	52 (55.3)	42 (55.3)	45 (54.9)
**Age (years)**			
Mean ± standard deviation	45 ± 13	45 ± 13	44 ± 13
Median, interquartile range	43, 35–53	42, 35–54	44, 35–53
**Tobacco**			
Active smokers	27 (28.7)	21 (27.6)	25 (30.5)
Non-active smokers	67 (71.3)	55 (72.4)	57 (69.5)
**Montreal Classification**			
A1	4 (4.3)	2 (2.6)	4 (4.9)
A2	65 (69.1)	52 (68.4)	56 (68.3)
A3	25 (26.6)	22 (28.9)	22 (26.8)
L1	45 (47.9)	37 (48.7)	40 (48.8)
L2	4 (4.2)	4 (5.3)	3 (3.7)
L3	45 (47.9)	35 (46.0)	39 (47.5)
B1	36 (38.3)	29 (38.1)	31 (37.8)
B2	41 (43.6)	35 (46.1)	35 (42.7)
B3	17 (18.1)	12 (15.8)	16 (19.5)
**Perineal disease**	11 (11.7)	2 (2.6)	11 (13.4)
**Upper gastrointestinal disease**	2 (1.88)	1 (1.31)	2 (2.43)
**Ileal resection**	24 (25.5)	21 (27.6)	21 (25.6)

**Table 2:** Vitamin B12 oral treatment regimens used for treatment of B12 deficiency.

Brand Name	Qualitative and Quantitative Composition	Dosage	Patients *n*
BENEXOL©	**B12 1 mg**, B6 250 mg, B1 250 mg	1 tablet/24 h	38
		1 tablet/12 h	6
		1 tablet/8 h	3
FOLIDOCE©	**B12 0.002 mg**, folic acid 0.4 mg	1 tablet/12 h	2
HIDROXIL©	**B12 0.5 mg**, B6 250 mg, B1 250 mg	1 tablet/24 h	1
NEURODAVUR©	**B12 2.5 mg**, B6 250 mg, B1 250 mg	1 tablet/24 h	3
NEUROMADE©	**B12 1 mg**, B6 50 mg, B1 50 mg	1 tablet/24 h	6
OPTOVITE©	**B12 1 mg**	1 ampule/24 h	9
		2 ampule/7 days	8

Benexol© (Bayer Hispania, Barcelona, Spain). Folidoce© (Italfarmaco España, Madrid, Spain). Hidroxil© (Almirall España, Barcelona, Spain). Neurdavur© (Davur laboratorios, Madrid, Spain). Neuromade (Teofarma, Barcelona, Spain). Optovite© (Normon, Madrid, Spain).

**Table 3:** Vitamin B12 oral treatment regimens used for maintenance treatment.

Brand Name	Qualitative and Quantitative Composition	Dosage	Patients *n*
BENEXOL©	**B12 1 mg**, B6 250 mg, B1 250 mg	1 tablet/24 h	22
		1 tablet/48 h	1
		2 tablet/7 days	17
FOLIDOCE©	**B12 0.002 mg**, folic acid 0.4 mg	1 tablet/12 h	2
NEURODAVUR©	**B12 2.5 mg**, B6 250 mg, B1 250 mg	1 tablet/24 h	8
NEUROMADE©	**B12 1 mg**, B6 50 mg, B1 50 mg	1 tablet/24 h	9
OPTOVITE©	**B12 1 mg**	2 ampule/7 days	23

Benexol© (Bayer Hispania, Barcelona, Spain). Folidoce© (Italfarmaco España, Madrid, Spain). Neurdavur© (Davur laboratorios, Madrid, Spain). Neuromade (Teofarma, Barcelona, Spain). Optovite© (Normon, Madrid, Spain).

**Table 4:** Serum cobalamin levels (pg/mL) before and after acute treatment with oral cyanocobalamin

**Patient**	**1**	**2**	**3**	**4**	**5**	**6**	**7**	**8**	**9**	**10**	**11**	**12**	**13**	**14**	**15**	**16**	**17**	**18**	**19**	**20**	**21**	**22**	**23**	**24**	**25**	**26**	**27**	**28**	**29**	**30**	**31**	**32**	**33**	**34**	**35**	**36**	**37**	**38**
Before	46	72	79	86	90	104	104	105	111	116	117	118	119	119	119	121	128	128	129	135	136	137	137	139	139	139	141	142	151	152	153	153	155	156	156	156	157	157
After	177	229	554	333	464	488	273	232	202	230	202	1369	622	375	233	446	222	247	452	402	367	313	436	305	251	394	219	203	198	779	187	294	627	204	228	360	803	562
**Patient**	**39**	**40**	**41**	**42**	**43**	**44**	**45**	**46**	**47**	**48**	**49**	**50**	**51**	**52**	**53**	**54**	**55**	**56**	**57**	**58**	**59**	**60**	**61**	**62**	**63**	**64**	**65**	**66**	**67**	**68**	**69**	**70**	**71**	**72**	**73**	**74**	**75**	**76**
Before	157	159	159	161	163	164	165	166	168	169	170	170	170	171	173	173	173	174	174	175	175	176	177	181	184	186	192	193	196	199	200	200	200	200	200	200	200	200
After	216	321	223	350	360	391	934	296	471	294	349	841	402	478	497	182	286	623	478	761	246	507	252	238	218	296	359	380	337	411	380	259	660	744	430	373	287	647

**Table 5:** Serum cobalamin levels (pg/mL) during maintenance therapy; at the beginning of follow-up and at the end of follow-up.

**Patient**	**1**	**2**	**3**	**4**	**5**	**6**	**7**	**8**	**9**	**10**	**11**	**12**	**13**	**14**	**15**	**16**	**17**	**18**	**19**	**20**	**21**	**22**	**23**	**24**	**25**	**26**	**27**	**28**	**29**	**30**	**31**	**32**	**33**	**34**	**35**	**36**	**37**	**38**	**39**	**40**	**41**
Beginning	202	202	202	204	214	216	218	222	223	228	229	230	232	238	244	246	247	251	252	259	273	286	287	294	294	296	296	304	305	305	313	321	337	338	349	350	359	360	360	364	367
End	168	255	155	271	214	422	211	295	310	193	201	224	289	491	288	209	413	263	176	174	303	279	287	294	194	463	372	359	233	166	186	176	283	316	454	293	359	468	270	364	215
**Patient**	**42**	**43**	**44**	**45**	**46**	**47**	**48**	**49**	**50**	**51**	**52**	**53**	**54**	**55**	**56**	**57**	**58**	**59**	**60**	**61**	**62**	**63**	**65**	**66**	**67**	**68**	**69**	**70**	**71**	**72**	**73**	**74**	**75**	**76**	**77**	**78**	**79**	**80**	**81**	**82**	
Beginning	369	371	373	375	380	380	389	391	394	402	411	430	432	436	446	452	464	471	478	488	489	497	532	554	560	587	622	623	645	647	660	695	744	761	803	841	885	934	1065	1500	
End	897	173	177	214	302	239	290	154	354	409	600	524	387	496	1304	177	239	229	365	709	398	460	167	535	496	413	416	391	494	729	440	522	328	351	1513	167	214	616	217	767	

## Supplementary Materials

The following are available online at http://www.mdpi.com/2072-6643/9/3/308/s1, Table S1: Serum cobalamin levels (pg/mL) and folic acid levels (ng/mL) before and after acute treatment with oral cyanocobalamin.
