# Supplementary material for: Oral Cyanocobalamin is Effective in the Treatment of Vitamin B12 Deficiency in Crohn’s Disease
Source: Nutrients. 2017 Mar 20;9(3):308. doi: 10.3390/nu9030308 (PMC5372971; doi:10.3390/nu9030308)
Supplement: Supplementary file 1 [file nutrients-09-00308-s001.docx]

**Supplementary Materials:** Oral Cyanocobalamin is Effective in the Treatment of Vitamin B12 Deficiency in Crohn's Disease

3. Results

3.1. Effectiveness of Oral Treatment in Vitamin B12 Deficiency

Seventy-six patients of the overall population included (94 patients) had vitamin B12 deficiency and were treated with oral B12 therapy to correct their deficiency. In these patients, the mean serum cobalamin concentration before oral treatment was 152.83 ± 34.09 pg/mL (median 157, interquartile range of 132–174.5 pg/mL). Seventy-two (94.7%) patients normalized their vitamin B12 levels (>200 pg/mL) after mean treatment duration of 193 ± 278 days (median 120, interquartile range 78–191). The mean serum cobalamin concentration after treatment increased to 398.14 ± 208.90 pg/mL (median 354.59, interquartile range: 242 to 474.50). Figure 1 shows the evident change of B12 levels and Table 4 cobalamin levels of each patient before and after treatment. In relation to hematologic manifestations of B12 deficiency, before oral B12 treatment, 19.7% (15) of patients had anemia (mean concentration of Hb: 13.61 ± 1.67), but no patient had macrocytic anemia. The erythrocyte MCV was elevated in 14 (18.4%) patients, although without anemia, with a mean of MCV of 91.54 ± 7.58. After treatment with oral vitamin B12, there were no significant changes in Hb concentration nor in MCV.

Folic acid levels (ng/mL), before and after treatment with oral cyanocobalamin, are shown in Supplementary Table 1 (Table S1).

**Table S1:** Serum cobalamin levels (pg/mL) and folic acid levels (ng/mL) before and after acute treatment with oral cyanocobalamin.

| **Patient** | **B12 levels before therapy** | **Folic acid levels before acute oral B12 therapy** | **B12 levels after therapy** | **Folic acid levels after acute oral B12 therapy** |
| --- | --- | --- | --- | --- |
| 1 | 46 | 3.7 | 177 | 4.0 |
| 2 | 72 | 5.0 | 229 | 4.6 |
| 3 | 79 | 1.7 | 554 | 13.9 |
| 4 | 86 | 8.7 | 333 | UK |
| 5 | 90 | 20.0 | 464 | 20.0 |
| 6 | 104 | 2.19 | 488 | 2 |
| 7 | 104 | 6.6 | 273 | 1.7 |
| 8 | 105 | 1.0 | 232 | 4.1 |
| 9 | 111 | 4.6 | 202 | 2.8 |
| 10 | 116 | 1.7 | 230 | 6.2 |
| 11 | 117 | 2.7 | 202 | 13.1 |
| 12 | 118 | 9.3 | 1369 | 3.6 |
| 13 | 119 | 2.8 | 622 | 4.0 |
| 14 | 119 | 3.84 | 375 | 4.3 |
| 15 | 119 | 9.84 | 233 | 3.9 |
| 16 | 121 | 7.7 | 446 | 7 |
| 17 | 128 | 4.43 | 222 | 6.9 |
| 18 | 128 | 6.5 | 247 | 8.3 |
| 19 | 129 | 3.6 | 452 | 3.9 |
| 20 | 135 | 2.9 | 402 | 2.7 |
| 21 | 136 | 2.9 | 367 | 4.1 |
| 22 | 137 | 1.5 | 313 | 3.7 |
| 23 | 137 | 3.3 | 436 | 4.3 |
| 24 | 139 | 2.9 | 305 | 3.7 |
| 25 | 139 | 3.9 | 251 | 6.9 |
| 26 | 139 | 4.0 | 394 | 8.0 |
| 27 | 141 | 5.4 | 219 | 5.8 |
| 28 | 142 | 4.7 | 203 | 3.5 |
| 29 | 151 | 8.7 | 198 | 5.4 |
| 30 | 152 | 4.1 | 779 | 2.3 |
| 31 | 153 | 3.4 | 187 | 3.4 |
| 32 | 153 | 8.6 | 294 | 9.1 |
| 33 | 155 | 6.8 | 627 | 4.7 |
| 34 | 156 | 4.0 | 204 | 1.9 |
| 35 | 156 | 4.6 | 228 | 2.4 |
| 36 | 156 | 5.7 | 360 | 5.4 |
| 36 | 157 | 2.5 | 803 | 2.5 |
| 38 | 157 | 4.6 | 562 | 9.1 |
| 39 | 157 | 5.9 | 216 | 4.7 |
| 40 | 159 | 3.26 | 321 | 1.8 |
| 41 | 159 | 3.3 | 223 | 3.7 |
| 42 | 161 | 5.2 | 350 | 2.8 |
| 43 | 163 | 11.0 | 360 | 6.0 |
| 44 | 164 | 2.2 | 391 | 1.9 |
| 45 | 165 | 4.1 | 934 | 1.0 |
| 46 | 166 | 4.4 | 296 | 3.5 |
| 47 | 168 | 8.6 | 471 | 15.3 |
| 48 | 169 | 2.0 | 294 | 3.9 |
| 49 | 170 | 2.1 | 349 | 9.4 |
| 50 | 170 | 4.9 | 841 | 2.1 |
| 51 | 170 | 4.6 | 402 | 3.2 |
| 52 | 171 | 3.1 | 478 | 2.4 |
| 53 | 173 | 4.6 | 497 | 3.0 |
| 54 | 173 | 11.5 | 182 | 9.5 |
| 55 | 173 | UK | 286 | UK |
| 56 | 174 | 1.8 | 623 | >20 |
| 57 | 174 | 3.0 | 478 | 2.8 |
| 58 | 175 | 4.1 | 761 | 3.7 |
| 59 | 175 | 7.4 | 246 | 7.5 |
| 60 | 176 | 3.8 | 507 | 2.2 |
| 61 | 177 | 5.5 | 252 | 3.7 |
| 62 | 181 | 1.0 | 238 | 20.0 |
| 63 | 184 | 2.2 | 218 | UK |
| 64 | 186 | 11.5 | 296 | 13.1 |
| 65 | 192 | 4.4 | 359 | 5.9 |
| 66 | 193 | 1.6 | 380 | 20.0 |
| 67 | 196 | 7.9 | 337 | 5.4 |
| 68 | 199 | UK | 411 | 4.1 |
| 69 | 200 | 1.8 | 380 | 2.0 |
| 70 | 200 | 2.7 | 259 | 3.5 |
| 71 | 200 | 3 | 660 | 1.9 |
| 72 | 200 | 3.2 | 744 | 1.8 |
| 73 | 200 | 4.7 | 430 | 4.9 |
| 74 | 200 | 5.3 | 373 | 3.3 |
| 75 | 200 | 5.3 | 287 | 8.3 |
| 76 | 200 | 6.4 | 647 | 6.7 |

UK: unknown
